# Encapsulation of SAAP-148 in Octenyl Succinic Anhydride-Modified Hyaluronic Acid Nanogels for Treatment of Skin Wound Infections

**DOI:** 10.3390/pharmaceutics15020429

**Published:** 2023-01-28

**Authors:** Miriam E. van Gent, Tom van Baaren, Sylvia N. Kłodzińska, Muhanad Ali, Natasja Dolezal, Bjorn R. van Doodewaerd, Erik Bos, Amy M. de Waal, Roman I. Koning, Jan Wouter Drijfhout, Hanne Mørck Nielsen, Peter H. Nibbering

**Affiliations:** 1 Department of Infectious Diseases, Leiden University Medical Center, 2300 RC Leiden, The Netherlands; 2 Center for Biopharmaceuticals and Biobarriers in Drug Delivery (BioDelivery), Department of Pharmacy, Faculty of Health and Medical Sciences, University of Copenhagen, DK-2100 Copenhagen, Denmark; 3 Department of Immunology, Leiden University Medical Center, 2300 RC Leiden, The Netherlands; 4 Department of Cell and Chemical Biology, Leiden University Medical Center, 2300 RC Leiden, The Netherlands; 5 Electron Microscopy Facility, Department of Cell and Chemical Biology, Leiden University Medical Center, 2300 RC Leiden, The Netherlands

**Keywords:** antimicrobial peptide, SAAP-148, hyaluronic acid-based nanogels, AMP formulation, skin wound infections, cutaneous application

## Abstract

Chronic wound infections colonized by bacteria are becoming more difficult to treat with current antibiotics due to the development of antimicrobial resistance (AMR) as well as biofilm and persister cell formation. Synthetic antibacterial and antibiofilm peptide (SAAP)-148 is an excellent alternative for treatment of such infections but suffers from limitations related to its cationic peptidic nature and thus instability and possible cytotoxicity, resulting in a narrow therapeutic window. Here, we evaluated SAAP-148 encapsulation in nanogels composed of octenyl succinic anhydride (OSA)-modified hyaluronic acid (HA) to circumvent these limitations. SAAP-148 was efficiently (>98%) encapsulated with high drug loading (23%), resulting in monodispersed anionic OSA-HA nanogels with sizes ranging 204–253 nm. Nanogel lyophilization in presence of polyvinyl alcohol maintained their sizes and morphology. SAAP-148 was sustainedly released from lyophilized nanogels (37–41% in 72 h) upon reconstitution. Lyophilized SAAP-148-loaded nanogels showed similar antimicrobial activity as SAAP-148 against planktonic and biofilm-residing AMR *Staphylococcus aureus* and *Acinetobacter baumannii*. Importantly, formulated SAAP-148 showed reduced cytotoxicity against human erythrocytes, primary human skin fibroblasts and human keratinocytes. Additionally, lyophilized SAAP-148-loaded nanogels eradicated AMR *S. aureus* and *A. baumannii* colonizing a 3D human epidermal model, without inducing any cytotoxicity in contrast to SAAP-148. These findings indicate that OSA-HA nanogels increase SAAP-148′s therapeutic potential for treatment of skin wound infections.

## 1. Introduction

Chronic wounds, such as diabetic foot ulcers and burn wounds, affect up to 3.5% of the United States population, constituting a painful burden for patients and a significant expenditure to healthcare systems and societies around the world [1,2,3]. As standard care, chronic wounds are regularly cleaned, debrided and covered using wound dressings [4]; however, the slow healing process of chronic wounds may allow bacteria to infiltrate and subsequently infect the wound. Opportunistic pathogens, such as *Staphylococcus aureus* and *Acinetobacter baumannii*, among others, are notorious for colonizing chronic wounds [5,6]. Moreover, antimicrobial resistance (AMR) development [7,8], biofilm formation [8] and the evolution of persister cells [9] by these pathogens hampers their effective eradication with current antibiotics. Therefore, there is an urgent need for novel and effective treatments of chronic wound infections.

Antimicrobial peptides (AMPs), such as synthetic antibacterial and antibiofilm peptide (SAAP)-148, are promising alternatives to antibiotics for treatment of bacterial wound infections. SAAP-148 is a potent and broad-spectrum antimicrobial that can eradicate AMR bacteria, including *S. aureus* and *A. baumannii* biofilms from murine skin [10]. Additionally, SAAP-148 is effective against AMR *S. aureus* persister cells in antibiotic-exposed mature biofilms [11]. Moreover, SAAP-148 has proven successful in the treatment of superficial skin wound infections in mice [10]. Nevertheless, SAAP-148 was not as successful in surgical skin wound infections in rats [12]. The limitations of SAAP-148 in the latter study were partly related to components within the wound micro-environment, such as binding to (plasma) proteins and degradation by proteases. Other challenges for further development of SAAP-148 are related to its cationic peptidic nature, including a short half-life and cytotoxicity, resulting in a narrow therapeutic window.

Nano-scaled drug delivery systems can be used to circumvent several of these limitations associated with SAAP-148. Drug delivery systems have been shown to improve pharmacokinetic and -dynamic properties of AMPs by (i) improving their stability and bioavailability [13,14,15], (ii) mediating their sustained release, thus reducing their overall cytotoxicity [16,17], (iii) assisting their transport across cellular membranes and improving intracellular uptake [17,18], and (iv) improving their biofilm penetration and intracellular retention [17,18,19,20]. In particular, nanogels are excellent carriers for wound treatment due to their combined features of nanoparticles and hydrogels, thereby allowing high encapsulation of water-soluble drugs, like AMPs, and providing a moist environment ideal for wound healing [21,22,23]. Nanogels composed of hyaluronic acid (HA) are of particular interest due to HA’s biodegradability, biocompatibility, and its intrinsic antiadhesive and antibiofilm properties towards bacteria [24]. Furthermore, modification of HA with octenyl succinic anhydride (OSA) produces an amphiphilic polymer [25], that can self-assemble into soft flexible nanogels composed of hydrophobic and hydrophilic zones within the nanogel matrix [22,26]. Finally, OSA-HA nanogels have been shown to reduce the cytotoxicity of a range of biomacromolecules, including AMPs [27], peptidomimetics [28], and antibiofilm peptides [16].

In this study, we encapsulated SAAP-148 in OSA-HA nanogels with the aim to improve its selectivity index. For this purpose, we determined the physicochemical properties of the nanogels and evaluated their antibacterial and cytotoxic activities in vitro and in a 3D human epidermal infection model. This study describes successful encapsulation of SAAP-148 in OSA-HA nanogels that showed reduced cytotoxicity and maintained antimicrobial activities against AMR bacteria, thus improving SAAP-148’s selectivity index.

## 2. Materials and Methods

### 2.1. Materials

Hyaluronic acid (HyaCare, 50 kDa) was purchased from Evonik Nutrition and Care (Essen, Germany). Octenyl succinic anhydride (OSA, 97% purity), lysozyme egg white, trifluoroacetic acid (TFA), tetracycline, Triton^TM^ X-100 and bovine serum albumin (BSA) were obtained from Sigma-Aldrich (St. Louis, MO, USA). Sodium bicarbonate (NaHCO_3_), sodium hydroxide (NaOH), calcium chloride (CaCl_2_) and human serum were obtained from Merck (Darmstadt, Germany). SAAP-148 was synthesized using standard Fmoc chemistry, purified to >95% as described previously [29], and stored lyophilized until use. 6-Carboxytetramethylrhodamine (6-TAMRA)-labeled SAAP-148 (acetyl-LK(6-TAMRA)RVWKRVFKLLKRYWRQLKKPVR-amide) with a purity of 96.9% was purchased from Pepscan (Lelystad the Netherlands). Polyvinyl alcohol (PVA) was purchased from Acros Organics (Geel, Belgium) and dextran-40 was a kind gift from Avivia B.V. (Nijmegen, the Netherlands). Uranyl acetate was obtained from Honeywell Fluka (Charlotte, NC, USA). Analytical-grade solvents for UPLC analysis included ultrapure water (Veolia Purelab Chorus 1, ELGA Labwater, High Wycombe, UK) and acetonitrile (100%, VWR, Radnor, PA, USA). Ultrapure water for synthesis of polymer, and sample preparation and analysis was obtained from a MilliPore system, and phosphate-buffered saline (PBS) from Fresenius Kabi (Graz, Austria). Tryptic soy broth (TSB), brain heart infusion (BHI) and Mueller–Hinton (MH) agar were purchased from Oxoid (Basingstoke, UK). Dulbecco’s Modified Eagle Medium (DMEM) supplemented with 1% (*v*/*v*) GlutaMAX™, penicillin-streptomycin (pen/strep), trypsin-EDTA, keratinocyte serum-free medium (KSFM), bovine pituitary extract (BPE) and human recombinant epidermal growth factor (EGF) were obtained from Gibco (Waltham, MA, USA). Microplates were purchased from Greiner BioOne (Alphen a/d Rijn, The Netherlands), and culture plates and inactivated fetal bovine serum (FBSi) from Corning Inc. (Corning, NY, USA). Alexa Fluor^TM^ Plus 405 Phalloidin, 4’,6-diamidino-2-fenylindool (DAPI) and ProLong Diamond Antifade mountant were purchased from Invitrogen (Waltham, MA, USA), while 1% (*v*/*v*) paraformaldehyde (PFA) was obtained from the Department of Clinical Pharmacy and Toxicology (Leiden University Medical Center, Leiden, The Netherlands).

### 2.2. Modification of Hyaluronic Acid with Octenyl Succinic Anhydride

Octenyl succinic anhydride-modified hyaluronic acid (OSA-HA, 15-20% degree of substitution, see Figures S1–S3) was synthesized as described previously [25]. In short, 1.25 g HA was dissolved in 50 mL ultrapure water and NaHCO_3_ was added and mixed for 1 h to yield a 2 M carbonate solution. Afterwards, the pH was adjusted to pH 8.5 with 0.5 M NaOH, and OSA was added dropwise to the HA solution to reach a molar OSA:HA ratio of 50:1. The solution was left to react overnight at room temperature. Then, the reaction product was dialyzed against ultrapure water at 4 °C until the conductivity reached 5 µS/cm and then lyophilized. The degree of substitution for OSA-HA was determined using ^1^H-NMR. The degree of substitution of OSA-HA, which corresponds to the number of grafted molecules per 100 disaccharide units was calculated by comparing the intensity per proton of the terminal methyl protons on the grafted octenyl succinate groups (0.9 ppm) to that of the methyl protons on native HA (2.0 ppm) [25].

### 2.3. Preparation of Octenyl Succinic Anhydride-Modified Hyaluronic Acid Nanogels

SAAP-148-loaded OSA-HA nanogels were produced at room temperature using a microfluidic chip design described previously [30]. Briefly, SAAP-148 and OSA-HA were dissolved in ultrapure water to a concentration of 1500 µg/mL (10× final peptide concentration) and 500 µg/mL, respectively. The solutions were filled into three gastight fixed Luer lock-tip glass syringes (Prosense, Oosterhout, The Netherlands) mounted on three NE-300 syringe pumps (Prosense) to control the flow rates. The OSA-HA solution was injected into the two outer streams of the microfluidic chip at a flow rate of 0.99 mL/min and the SAAP-148 solution was injected in the inner stream at a flow rate of 0.22 mL/min, resulting a combined flow of 2.2 mL/min. The produced nanogels contained 150 µg/mL SAAP-148 and 500 µg/mL OSA-HA. Freshly prepared nanogels and lyophilized and redispersed nanogels were used, and the concentration of peptide is given as the total peptide concentration present in the sample. Freshly prepared nanogels were stored at 4 °C and used within one week. For lyophilization of nanogels, 1–10 mg/mL PVA or dextran-40 was added in a 1:1 (*v*/*v*) ratio to the nanogels. Subsequently, the nanogel solution was frozen with liquid N_2_ and lyophilized overnight at −52 °C and 0.025 mbar using an Alpha 1–4 LSCbasic freeze dryer (Martin Christ Gefriertrocknungsanlagen, Osterode am Harz, Germany) with RV3 vacuum pump and EMF10 oil mist filter (Edwards, Burgers Hill, UK). Freshly prepared nanogels were diluted and lyophilized nanogels were redispersed in the relevant media before use.

### 2.4. Physicochemical Properties of OSA-HA Nanogels

The average size, polydispersity index (PDI) and zeta potential (ZP) of the OSA-HA nanogels was determined using dynamic light scattering. The size, PDI and ZP measurements of OSA-HA nanogels were performed at a concentration of 150 µg/mL SAAP-148 and/or 500 µg/mL OSA-HA in ultrapure water at 25 °C using a Zetasizer Nano ZS (Malvern Instruments, Worcestershire, UK) equipped with a 633 nm laser and 173° detection optics. Malvern DTS v.6.20 software was used for data acquisition and analysis. Measurements were performed in triplicate for at least three independent sample batch replicates.

### 2.5. Transmission Electron Microscopy

OSA-HA nanogels were visualized using negative stain transmission electron microscopy (TEM). In short, 200 mesh formvar and carbon coated copper EM grids (Agar Scientific, Stansted, UK) were glow-discharged by 0.2 mbar air for 1 min using the glow discharger unit of an EMITECH K950X (Quorum Technologies, Lewes, UK). Three µL of OSA-HA nanogel solution (containing 150 µg/mL SAAP-148 and 500 µg/mL OSA-HA) was applied per glow-discharged grid for 1 min and the grids were blotted to remove excess of sample. Subsequently, the grids were stained on droplets of 2% (*w*/*v*) uranyl acetate in water for 1 min, after which excess staining solution was removed with blotting paper. Imaging of the air-dried grids was performed at 120 kV on a Tecnai 12 electron microscope (ThermoFisher, Waltham, MA, USA). A 4k × 4k Eagle camera (ThermoFisher) was used to record images at 11,000× magnification.

### 2.6. Quantification of SAAP-148

Quantification of SAAP-148 was performed using an ACQUITY H-class UPLC-MS system (Waters, Milford, MA, USA) with an LCT-premier mass detector (Waters). Chromatographic separation was carried out using an ACQUITY UPLC BEH C18 column (100 × 2.1 mm, 1.7 µm; Waters). The mobile phase consisted of eluent A (100% ultrapure water) and eluent B (100% acetonitrile), both containing 0.05% (*v*/*v*) TFA. Samples were run with a gradient of 5 to 75% eluent B over 8 min at 0.5 mL/min at 50 °C. The data were analyzed with respect to a calibration curve of SAAP-148 (0.01–0.1 mg/mL) using MassLynx Software V4.2.

### 2.7. Encapsulation Efficiency and Drug Loading of SAAP-148 in OSA-HA Nanogels

The amount of encapsulated SAAP-148 in the OSA-HA nanogels was determined indirectly by measuring the residual amount of peptide present in the aqueous bulk phase after nanogel production. The aqueous bulk phase was obtained by centrifuging the nanogels at 500,000× *g* for 30 min to ensure sedimentation of the nanogels. Quantification of encapsulation efficiency (EE) was performed in triplicate for three independent sample batch replicates. The calculations of the EE are based on the theoretical drug loading, because only non-encapsulated SAAP-148 could be measured:

(1) $$\mathrm{EE}\;\left(\%\right)=\frac{\mathrm{Total}\;\mathrm{SAAP}-148\;\left(µg\right)-\mathrm{Unencapsulated}\;\mathrm{SAAP}-148\;\left(µg\right)}{\mathrm{Total}\;\mathrm{SAAP}-148\;\left(µg\right)}\times 100\%$$

The drug loading (DL) is calculated similarly:

(2) $$\mathrm{DL}\;\left(\%\right)=\frac{\mathrm{Encapsulated}\;\mathrm{SAAP}-148\;\left(µg\right)}{\mathrm{OSA}-\mathrm{HA}\;\mathrm{polymer}\;\left(µg\right)+\mathrm{Encapsulated}\;\mathrm{SAAP}-148\;\left(µg\right)}\times 100\%$$

### 2.8. Release of SAAP-148 from PVA-Lyophilized OSA-HA Nanogels

In vitro release studies with PVA-lyophilized and redispersed SAAP-148-loaded nanogels were performed in PBS using dialysis membranes (Spectra-Por^®^ Float-a-Lyzer^®^ G2, MWCO 100 kDa, Spectrum Labs, Breda, The Netherlands). Prior to use, dialysis membranes were soaked and washed according to the manufacturer’s protocol to remove all present salts. Next, dialysis membranes were incubated with 1 mg/mL lysozyme egg white for 1 h at 37 °C and 200 rpm using an Innova^®^ 40/40R orbital shaker (New Bunswick Scientific, Nijmegen, The Netherlands) to reduce binding of peptide to the membrane. Dialysis membranes were washed with ultrapure water and 1 mL of SAAP-148 or SAAP-148-loaded nanogels (300 µg/mL) were loaded inside the dialysis cassette and placed in 6 mL of PBS while continuously shaking at 200 rpm. The temperature was maintained at 37 °C throughout the experiment and 1 mL samples were taken until 5 h and sample volume was replaced by equal volume of PBS. From 5 h onwards, 6 mL samples were taken. Samples were stored at −20 °C until analysis by UPLC. Results are expressed as percentage of SAAP-148 released from the nanogels normalized to diffusion of SAAP-148 solution as a control.

Alternatively, release of SAAP-148 from nanogels was determined by a centrifugation method, where PVA-lyophilized SAAP-148-loaded nanogels were redispersed in 1 mL PBS to a concentration of 150 µg/mL, and these samples were continuously shaken at 37 °C and 200 rpm. The vials were centrifuged at 500,000× *g* for 30 min at different time points, the supernatants collected and stored at −20 °C until analysis by UPLC. Results are expressed as SAAP-148 released from the nanogels relative to theoretical total amount of SAAP-148 loaded in the nanogels.

### 2.9. Bacteria

In this study, AMR strains of *S. aureus* (LUH14616; NCCB100829) and *A. baumannii* (RUH875), and GFP-producing methicillin-resistant *S. aureus* (MRSA; USA300 JE2) were used. Bacteria were stored in glycerol at −80 °C until use. Prior to experiments, non-GFP producing bacteria were cultured overnight on blood agar plates (BioMérieux SA, Marcy-l’Étoile, France) at 37 °C. On the day of the experiment, 3–5 bacterial colonies were cultured to mid-log phase in 10 mL TSB or BHI for 2.5 h at 37 °C while shaking at 200 rpm in an orbital shaker. Afterwards, bacteria were centrifuged at 1000× *g* for 10 min, washed with PBS, and resuspended in the preferred medium to the required concentrations based on the optical density at 600 nm. On the contrary, GFP-producing MRSA was cultured overnight in TSB containing 5 µg/mL tetracycline at 37 °C while shaking at 200 rpm in an orbital shaker. On the day of the experiment, the bacterial culture was split 1:333 (*v*/*v*) and grown for an additional 2.5 h to mid-log phase at 37 °C before washing steps and optical density measurements were performed.

### 2.10. In Vitro Killing Assay

Mid-log phase bacteria were resuspended in PBS to a concentration of 5 × 10^6^ colony forming units (CFU)/mL. Next, 20 µL bacterial suspension was mixed with 30 µL PBS containing increasing concentrations of SAAP-148, PVA-lyophilized and redispersed SAAP-148-loaded nanogels or placebo nanogels and 50 µL of filtered, inactivated and centrifuged human plasma (Sanquin, Leiden, The Netherlands) in polypropylene V-shaped 96-well microplates. After incubation for 4 h or 24 h at 37 °C under rotation at 200 rpm using an orbital shaker, 10-fold serial dilutes were made and plated onto MH agar plates to determine the number of viable bacteria. Results are expressed as lethal concentration (LC)_99.9_, i.e., the lowest concentration of SAAP-148 killing 99.9% of the inoculum.

### 2.11. In Vitro Biofilm Breakdown Assay

Mid-log phase bacteria were diluted to 1 × 10^7^ CFU/mL in BHI to grow 24 h biofilms. Briefly, 100 µL of bacterial suspension was added to each well of a polypropylene flat-bottom microplate and the plates were incubated for 24 h at 37 °C in a humidified environment. The next day, planktonic bacteria were removed from the wells and the biofilms were washed twice with PBS to remove non-adherent bacteria. Then, biofilms were exposed to PBS containing increasing concentrations of SAAP-148, PVA-lyophilized and redispersed SAAP-148-loaded nanogels or placebo nanogels. The plates were sealed with non-breathable plastic film sealers (Amplistar adhesive plate sealers, Westburg, Leusden, the Netherlands) and incubated for 24 h at 37 °C under continuous rotation using a shaking incubator. Medium controls were used to monitor possible contamination. Finally, the biofilms were washed twice with PBS and the biofilm-residing bacteria were harvested in 100 µL PBS by sonication (40 kHz, 10 min) using a Branson 1800 sonicator (Branson Ultrasonics BV, Ede, The Netherlands). The number of viable bacteria was assessed microbiologically. Results are expressed as biofilm eradication concentration (BEC)_99.9_, i.e., the lowest concentration of SAAP-148 that killed 99.9% of the biofilm-encased bacteria.

### 2.12. Hemolysis Assay

Whole blood from healthy donors (Sanquin, NVTO128.02, with informed consent) was collected in citrate tubes (BD Vacutainer Systems, Plymouth, UK), centrifuged at 1811× *g* to pellet the erythrocytes, washed three times using PBS and diluted in PBS to a 2% (*v*/*v*) erythrocyte suspension. Next, 25 µL of PBS containing increasing concentrations of SAAP-148, lyophilized and redispersed SAAP-148-loaded nanogels or placebo nanogels were mixed with 50 µL of pooled human plasma or PBS and 25 µL of 2% (*v*/*v*) human erythrocytes in wells of a polypropylene V-shaped microplate. A 5% (*v*/*v*) Triton^TM^ X-100 solution in PBS and PBS solution were included as positive and negative control, respectively. The plate was incubated for 1 h at 37 °C and 5% CO_2_, after which the erythrocytes were pelleted by centrifugation for 3 min at 290× *g*. The supernatant was transferred to a 96-well flat-bottom plate and the optical density was measured at 415 nm. Calculations of hemolytic activity are based on the following formula:

(3) $$\mathrm{Hemolysis}\;\left(\%\right)=\frac{\mathrm{OD}415_{\mathrm{sample}}-\mathrm{OD}415_{\mathrm{negative}\;\mathrm{control}}}{\mathrm{OD}415_{\mathrm{positive}\;\mathrm{control}}-\mathrm{OD}415_{\mathrm{negative}\;\mathrm{control}}}\times 100\%$$

Results are expressed as effective concentration (EC)_50_, i.e., the concentration of SAAP-148 resulting in 50% hemolysis. Non-linear regression curves with bottom and top restrictions at 0 an 100% were fit to each individual experiment to determine the medians (and ranges) of the EC_50_ values.

### 2.13. Cytotoxicity Assays Using Human Primary Skin Fibroblasts and Human Ker-CT Keratinocytes

Human primary skin fibroblasts (kindly provided by Dr. A. El Ghalbzouri, Department of Dermatology, LUMC) were cultured in culture flasks using DMEM supplemented with 1% (*v*/*v*) GlutaMAX™, 1% (*v*/*v*) pen/strep and 5% (*v*/*v*) FBSi. Next, fibroblasts were harvested using 0.05% (*w*/*v*) trypsin-EDTA, washed and resuspended to 2 × 10^5^ cells/mL in DMEM supplemented with 1% (*v*/*v*) GlutaMAX, 1% (*v*/*v*) pen/strep and 0.5% (*v*/*v*) human serum. Human keratinocytes of the Ker-CT cell line (ATCC^®^ CRL-4048™, Manassas, VA, USA) were cultured in culture flasks using KSFM supplemented with BPE, EGF, 0.3 M CaCl_2_ and 1% (*v*/*v*) pen/strep. The keratinocytes were harvested using trypsin-EDTA, washed and resuspended to 2 × 10^5^ cells/mL in their culture medium. For both cell types, 20,000 cells were seeded in 96-well culture plates and monolayers formed overnight at 37 °C and 5% CO_2_. Monolayers were exposed for 4 h or 24 h to increasing concentrations of SAAP-148, PVA-lyophilized and redispersed SAAP-148-loaded nanogels or placebo nanogels dissolved in the respective seeding medium. 1% (*v*/*v*) Triton^TM^ X-100 was used as positive control and medium as negative control. Lactate dehydrogenase (LDH) release from dead cells to the supernatants was detected by the Cytotoxicity Detection Kit (Roche, Basel, Switzerland) and the metabolic activity of the cells was assessed using cell proliferation reagent WST-1 (Roche), both according to the manufacturer’s instructions. Results are expressed as EC_50_ and non-linear regression curves with bottom and top restrictions at 0 and 100% were fit to each individual experiment to determine the medians (and ranges) of the EC_50_ values.

### 2.14. 3D Human Epidermal Infection Model

Human skin equivalents (HSEs) were cultured over 14 days using Ker-CT cells as previously described in detail [29]. At least two days before infection, their culture medium was replaced for culture medium without antibiotics. The HSEs were infected with AMR *S. aureus* or *A. baumannii* at a concentration of 1 × 10^5^ CFU/model for 1 h at 37 °C and 5% CO_2_. After infection, the bacterial suspension was removed, the cells were washed with PBS and the HSEs were treated with SAAP-148, PVA-lyophilized and redispersed SAAP-148-loaded nanogels or placebo nanogels at the desired concentrations in PBS for 4 h after which the supernatants (non-adherent bacteria) were stored on ice, while the HSEs (adherent bacteria) were homogenized using a bead-beater and both fractions were microbiologically assessed. Results are expressed as individual values and medians of three individual measurements performed in duplicate.

### 2.15. Cytotoxicity Assays in a 3D Human Epidermal Model

HSEs were exposed to SAAP-148, lyophilized and redispersed SAAP-148-loaded nanogels or placebo nanogels at the desired concentrations in PBS for 4 h including 1% (*v*/*v*) Triton^TM^ X-100 as positive control and PBS as negative control. Afterwards, LDH release from dead cells to the basal medium was detected by the Cytotoxicity Detection Kit according to manufacturer’s instructions. Furthermore, the HSEs were cut out, transferred to 24-well flat-bottom culture plates, and exposed to WST-1 reagent in DMEM medium overnight to determine the metabolic activity of the cells in the models. Read-out of medium solutions without the HSEs was performed according to manufacturer’s protocol. Results are expressed as percentage cytotoxicity or metabolic activity relative to controls.

### 2.16. Confocal Microscopy of an MRSA-Colonized 3D Human Epidermal Model

HSEs were changed to antibiotic-free medium at least two days before infection with GFP-producing MRSA. Bacteria were added to the HSEs at a concentration of 1 × 10^8^ CFU/model, spun down on top of the HSEs for 2 min at 300× *g*, and incubated for 1 h at 37 °C and 5% CO_2_ to infect the HSEs. After infection, the bacterial suspension was removed, and the HSEs were treated with TAMRA-SAAP-148 or PVA-lyophilized and redispersed TAMRA-SAAP-148-loaded nanogels in PBS for 4 h after which the HSEs were fixed in 1% (*v*/*v*) PFA for 1 h at 4 °C. HSEs were washed and stored in PBS at 4 °C until staining. Cell culture inserts were blocked with PBS containing 1% (*w*/*v*) BSA and 0.3% (*v*/*v*) Triton^TM^ X-100 (PBT) for 15 min at room temperature. Transparent membranes were removed from plastic inserts with a scalpel and the membranes were incubated with Phalloidin Alexa Fluor 405 diluted 1:50 (*v*/*v*) and DAPI diluted 1:100 (*v*/*v*) in PBT for 2 h at 4 °C. Membranes were washed three times in PBS and three times in demineralized water, then placed on a glass slide, and treated with ProLong Diamond Antifade mountant. All samples were imaged with a Leica SP-8 upright confocal microscope and 3D images were produced using a Leica SP8 WLL-2 inverted confocal microscope (Leica Microsystems, Wetzlar, Germany).

### 2.17. Statistics

Statistical differences among groups were evaluated by a Kruskal–Wallis test, followed by a Mann–Whitney rank sum test using Graphpad Prism software version 6.0 (Graph Pad Software, San Diego, CA, USA). Differences were considered statistically significant when *p* < 0.05.

## 3. Results

### 3.1. Physicochemical Properties of Freshly Produced OSA-HA Nanogels Containing SAAP-148

Microfluidics were used to mix increasing concentrations of SAAP-148 with OSA-HA in a fixed ratio to determine the optimal loading conditions. Results revealed very efficient encapsulation of SAAP-148 in these nanogels, with low polydispersity and anionic surface charge (Table 1). Increasing the concentration of SAAP-148 significantly (*p* = 0.0021) increased particles sizes from 229 nm to 419 nm. OSA-HA nanogels containing 150 µg/mL SAAP-148 were selected for further evaluation, since a particle size between 5 to 100–200 nm, but not exceeding 500 nm, is preferred for targeting bacterial biofilms and to prevent clearance from the blood stream by the complement system [31,32].

### 3.2. Effect of Lyophilization on the Physicochemical Properties and Morphology of SAAP-148-Loaded OSA-HA Nanogels

**Figure 1:**
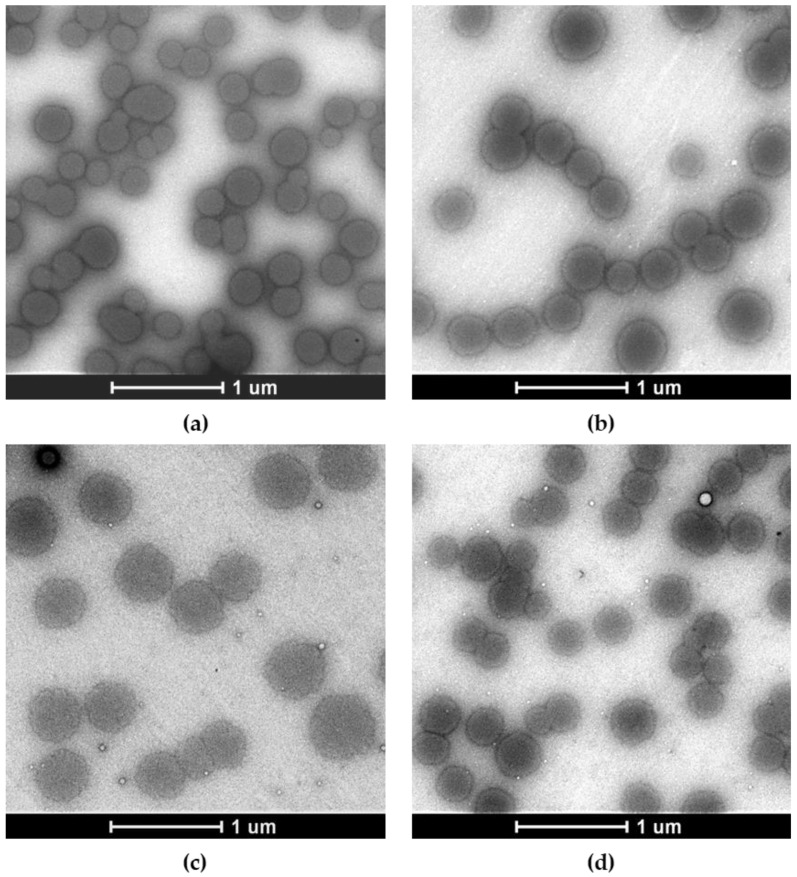
Representative negative stain transmission electron microscopy images of freshly produced SAAP-148-loaded and placebo OSA-HA nanogels compared to redispersed nanogels upon lyophilization in presence of 10 mg/mL PVA. The effect of lyophilization with 10 mg/mL PVA on visual properties of these nanogels was investigated. Shown are images of (**a**) freshly prepared SAAP-148-loaded nanogels, (**b**) lyophilized and redispersed SAAP-148-loaded nanogels, (**c**) freshly prepared placebo nanogels, and (**d**) lyophilized and redispersed placebo nanogels. Scale bar = 1 µm.

Lyophilization is necessary for concentrating the OSA-HA nanogels and for their long-term storage. A lyophilization study with 1–10 mg/mL PVA and dextran-40 was performed to select the optimal conditions for lyophilization of the SAAP-148-loaded nanogels. PVA and dextran-40 were selected based on their previous success in lyophilization of OSA-HA nanogels containing Ab-Cath [van Gent and Nibbering, personal communication]. Results revealed that lyophilization without cryoprotectants increased the size (*p* = 0.0021) and polydispersity (*p* = 0.0021) of SAAP-148-loaded nanogels upon redispersion in ultrapure water, while the encapsulation efficiency was also lowered by 18% (*p* = 0.0091), resulting in more negatively charged nanogels (*p* = 0.0010) with a ZP of –24.4 mV (Table 2). Lyophilization in the presence of 10 mg/mL PVA maintained the size of SAAP-148-loaded nanogels, while minimally increasing the polydispersity (*p* = 0.021) and only slightly reducing the peptide EE and DL to 93.4% (*p* = 0.0091), and 21.9% (*p* = 0.0045), respectively. As expected, the ZP of the nanogels was significantly (*p* = 0.0010) lowered in the presence of the anionic PVA. Lower concentrations of PVA and 1–10 mg/mL of dextran-40 were not able to protect SAAP-148-loaded nanogels during lyophilization. Additionally, morphology of freshly prepared SAAP-148-loaded and placebo nanogels were compared to these nanogels lyophilized in presence of 10 mg/mL PVA using transmission electron microscopy. Results revealed monodispersed spherical nanogels and confirmed that PVA stabilized the nanogels during the lyophilization process and maintained the morphology of the nanogels (Figure 1). Together, these data indicate that PVA at a concentration of 10 mg/mL protects SAAP-148-loaded nanogels during lyophilization, thus 10 mg/mL PVA-lyophilized SAAP-148-loaded nanogels were selected for further evaluation.

### 3.3. Sustained Release of SAAP-148 from PVA-Lyophilized OSA-HA Nanogels

**Figure 2:**
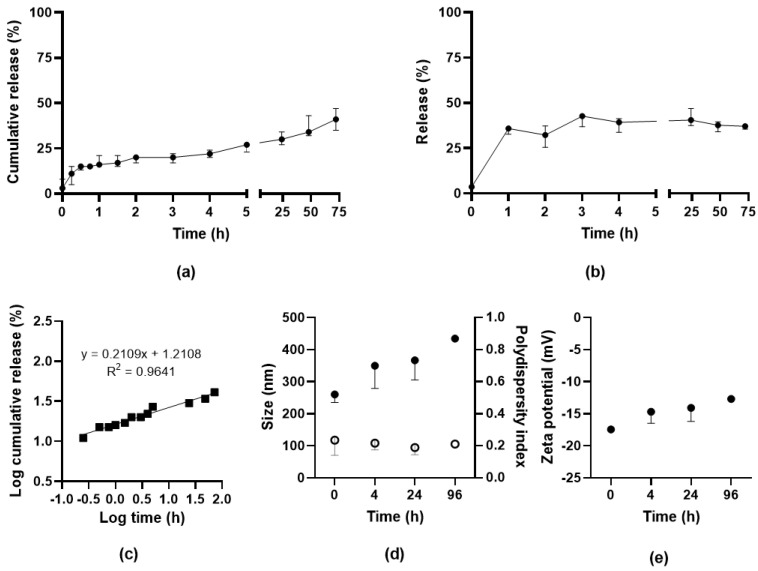
Release of SAAP-148 from PVA-lyophilized OSA-HA nanogels. Release of SAAP-148 from lyophilized OSA-HA nanogels in PBS at 37 °C using (**a**) dialysis method or (**b**) centrifugation method. Results are shown as median and range of three independent experiments and percentages are relative to the theoretical amount loaded. The Korsmeyer–Peppas model was applied to the logarithmic medians of the cumulative release (**c**). Effect of redispersion on (**d**) size (filled) and polydispersity index (open) and (**e**) zeta potential of PVA-lyophilized SAAP-148-loaded nanogels. Results are shown as median and range of three independent experiments, except for the 96 h time point that was performed once.

The release of SAAP-148 from OSA-HA nanogels redispersed in PBS after lyophilization with 10 mg/mL PVA was evaluated at 37 °C over a time period of 72 h by two methods, namely dialysis and centrifugation. Results from the dialysis study showed that SAAP-148 was released from the nanogels in biorelevant medium with a burst release of 16% in the first hour followed by gradual release up to 41% within 72 h, indicating a sustained release of SAAP-148 from OSA-HA nanogels over multiple days (Figure 2a). As a control, it was shown that diffusion of SAAP-148 in solution was slowed by the dialysis membrane, but all peptide was recovered within 72 h (Figure S4). Evaluation of the release-exponent (n) of the median percentages of SAAP-148 cumulatively released from the OSA-HA nanogels using the Korsmeyer–Peppas model revealed that n = 0.2109 with R^2^ = 0.9641 (Figure 2b). This n-value is below 0.5, and therefore suggests that the mechanism of drug release followed a quasi-Fickian process [33], indicating partial diffusion and controlled release of SAAP-148 from the OSA-HA nanogels. The centrifugation method confirmed the burst release of SAAP-148 with 36% of SAAP-148 released in the first hour with no further release for up to 72 h (Figure 2c). Both methods indicated that SAAP-148 was released to a limited extend from the nanogels, therefore the swelling of the nanogels in PBS was evaluated. Results showed that the nanogels expand over time, more than doubling in size within 4 h, but not resulting in complete dissociation of the nanogels withing 96 h (Figure 2d). This size expansion is combined with a slow increase in outer surface charge over time (Figure 2e).

### 3.4. Antimicrobial Activities of PVA-Lyophilized SAAP-148 OSA-HA Nanogels

The antimicrobial activities of SAAP-148-loaded nanogels redispersed in PBS after lyophilization in presence of 10 mg/mL PVA were compared to SAAP-148 against planktonic and biofilm-residing AMR bacteria. Results revealed that SAAP-148-loaded nanogels were 2-fold less effective in killing planktonic AMR *S. aureus* and *A. baumannii* after 4 h exposure compared to SAAP-148 in PBS containing 50% (*v*/*v*) plasma; however, after 24 h of exposure the antimicrobial activities were identical (Table 3). In addition, SAAP-148-loaded nanogels equally well eradicated AMR *S. aureus* biofilms in 24 h but were slightly less effective against AMR *A. baumannii* biofilms compared to SAAP-148 in PBS. Placebo nanogels did not show antibacterial and antibiofilm activities.

### 3.5. Hemolytic and Cytotoxic Activities of PVA-Lyophilized SAAP-148 OSA-HA Nanogels

Next, the hemolytic and cytotoxic activities of SAAP-148-loaded nanogels redispersed in the relevant medium after lyophilization using 10 mg/mL PVA were compared to SAAP-148. Results revealed that encapsulation of SAAP-148 in OSA-HA nanogels decreased the peptide’s hemolytic activity 1.9–2.5-fold and reduced the cytotoxic activities 2.9–3.6-fold and 1.5–2.3-fold for human primary skin fibroblasts and Ker-CT keratinocytes, respectively (Table 4). Moreover, the hemolytic activity of SAAP-148 was reduced dramatically in presence of 50% plasma and the cytotoxic activities of SAAP-148 to human primary skin fibroblasts and Ker-CT keratinocytes were slightly increased with time. Together, these data indicate that encapsulation of SAAP-148 in OSA-HA nanogels reduced the peptide’s hemolytic and cytotoxic activities >2-fold.

### 3.6. SAAP-148-Loaded OSA-HA Nanogels Protected with PVA during Lyophilization Effectively Eradicate AMR S. aureus and A. baumannii Infections from a 3D Human Epidermal Model

**Figure 3:**
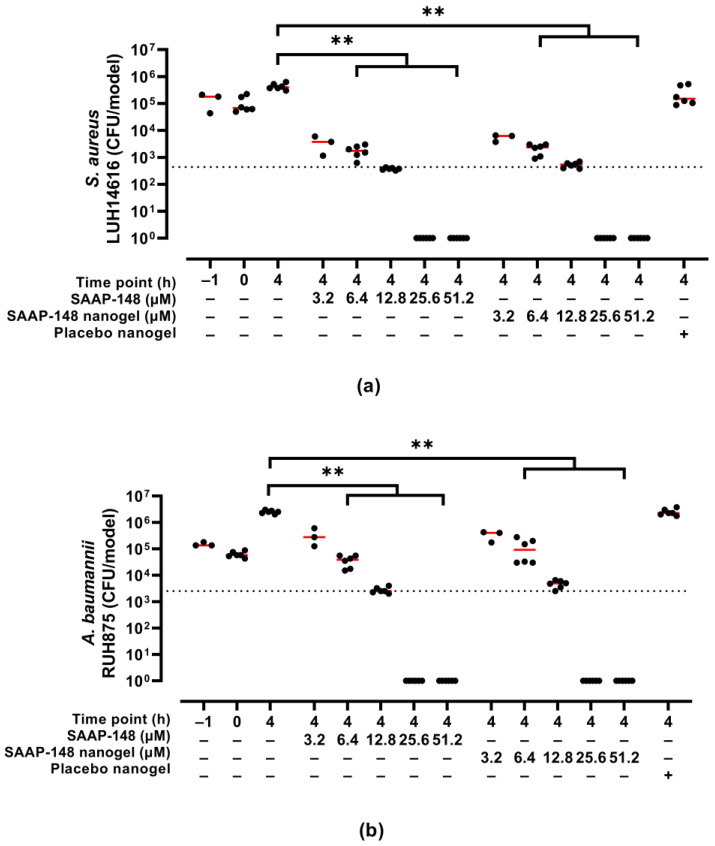
Bactericidal effects of redispersed PVA-lyophilized SAAP-148-loaded nanogels to colonized 3D human epidermal models compared to SAAP-148 and placebo nanogels. The 3D human epidermal model was infected for 1 h with AMR (**a**) *S. aureus* or (**b**) *A. baumannii* before exposure for 4 h to SAAP-148, SAAP-148 nanogel or placebo nanogel. The amount of placebo nanogel was equal to that of the highest dose of SAAP-148 nanogel. Data are shown as median and individual values of three experiments performed in singlicate or duplicate. Statistical differences between two groups are depicted as ** for *p* ≤ 0.01. The dashed line indicates the LC_99.9_, i.e., the lowest SAAP-148 concentration required to eradicate 99.9% of the bacteria colonizing the model.

The efficacy of SAAP-148-loaded nanogels upon reconstitution in PBS after lyophilization using 10 mg/mL PVA was evaluated in a 3D human epidermal model colonized with AMR *S. aureus* or *A. baumannii*. Results revealed that SAAP-148-loaded nanogels dose-dependently eradicated both bacterial strains colonizing the 3D human epidermal model to the same extent as SAAP-148 in solution (Figure 3). Exposure of the infected 3D human epidermal model to the highest dose of placebo nanogels was without antibacterial effect.

### 3.7. SAAP-148-Loaded OSA-HA Nanogels Protected with PVA during Lyophilization Reduce Cytotoxic Activities of the Peptide in a 3D Human Epidermal Model

**Figure 4:**
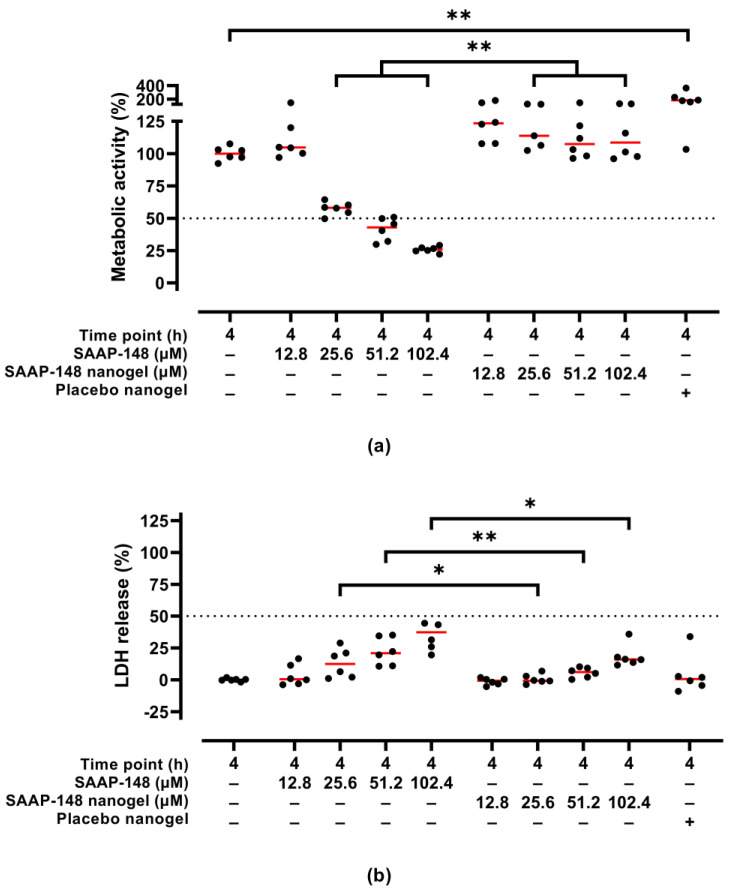
Cytotoxic activities of redispersed PVA-lyophilized SAAP-148-loaded nanogels to a 3D human epidermal model compared to SAAP-148 and placebo nanogels. The 3D human epidermal model was exposed for 4 h to SAAP-148, SAAP-148 nanogel or placebo nanogel in PBS. The cells in the model were evaluated based on (**a**) metabolic activity using WST-1 and (**b**) LDH release to the basal medium as measure for membrane disruption and/or perturbation. The amount of placebo nanogel was equal to that of the highest dose of SAAP-148 nanogel. Data are shown as median percentage and individual percentages of three experiments performed in duplicate relative to 1% (*v*/*v*) Triton^TM^ X-100 and PBS as positive and negative control, respectively. Statistical differences between two groups are depicted as * for *p* ≤ 0.05 and ** for *p* ≤ 0.01. The dashed line indicates the EC_50_, i.e., the SAAP-148 concentration resulting in 50% metabolic activity or 50% LDH release of the cells in the model.

Moreover, the cytotoxic activities of SAAP-148-loaded nanogels upon reconstitution in PBS after lyophilization using 10 mg/mL PVA were evaluated in a 3D human epidermal model. Results revealed that SAAP-148 in PBS dose-dependently reduced the metabolic activity of the eukaryotes in the model and simultaneously increased LDH release from these cells to the basal medium, while encapsulation of SAAP-148 in OSA-HA nanogels lyophilized with 10 mg/mL PVA prevented a reduction in metabolic activity of the cells in the models and reduced LDH release from these cells (Figure 4). Unexpectedly, placebo nanogels increased the metabolic activity of the cells in the model, but did not have any effect on the LDH release from these cells.

### 3.8. TAMRA-SAAP-148-Loaded OSA-HA Nanogels Protected with PVA during Lyophilization Penetrate into the Superficial Layers of a 3D Human Epidermal Model

**Figure 5:**
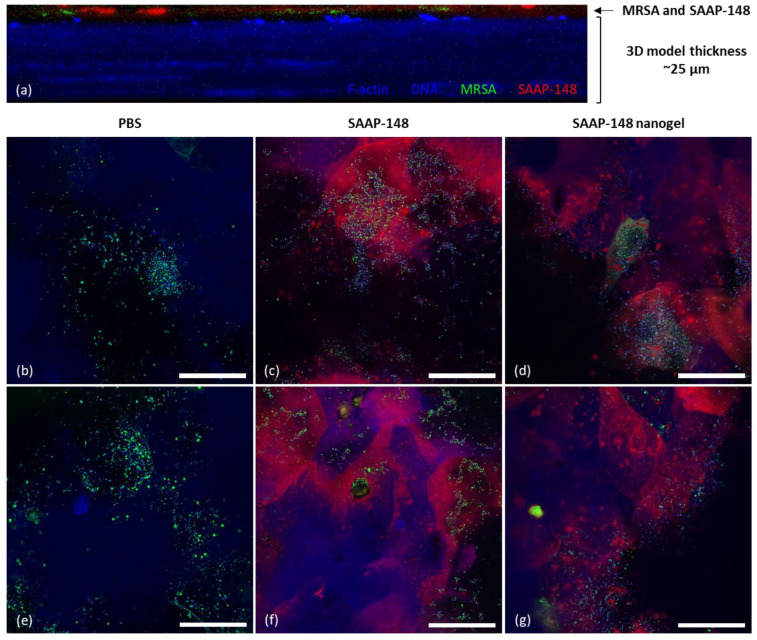
The penetration capabilities of TAMRA-SAAP-148-loaded nanogels compared to TAMRA-SAAP-148 in a 3D human epidermal model colonized with GFP-producing MRSA. Fluorescence confocal microscopy of a 3D human epidermal model colonized with GFP-producing MRSA (green) and treated for 4 h with 5 µM TAMRA-SAAP-148 (red) in PBS or 5 µM TAMRA-SAAP-148 nanogels (red) redispersed in PBS after lyophilization with 10 mg/mL PVA. Samples were stained for filamentous actin (F-actin) using Alexa Fluor 405 (blue) and with 4′,6-diamidino-2-phenylindole (DAPI) DNA staining (blue). (**a**) Cross-sectional image of the MRSA-colonized HSE model treated with TAMRA-SAAP-148-loaded nanogels indicating that MRSA and TAMRA-SAAP-148-loaded nanogels localize in the top layers of the model. Images in (**b**,**e**) demonstrated colonization of the GFP-producing MRSA on the 3D human epidermal model when treated with PBS, while images of the MRSA-colonized model treated with TAMRA-SAAP-148 are shown in (**c**,**f**) and with TAMRA-SAAP-148 nanogel treatment in (**d**,**g**). Images were taken with a 63× oil lens and are shown as representative images of two experiments performed in duplicate. Scale bar = 50 µm. Freshly produced TAMRA-SAAP-148 nanogels were checked for their physicochemical properties and shown to be 158 nm in size with a PDI of 0.18 and a ZP of –24.7 mV (n = 1).

Finally, confocal microscopy was used to image the 3D human epidermal model colonized with GFP-producing MRSA and exposed to TAMRA-SAAP-148-loaded nanogels or TAMRA-SAAP-148 to analyze their penetration capability into the cell layers of this model. It was shown that the 3D human epidermal model had a thickness of about 25 µm, similar to the non-colonized model (Figure S5), and that MRSA and TAMRA-SAAP-148-loaded nanogels localize only in the superficial layers of the 3D human epidermal model upon 4 h incubation (Figure 5a). Notably, labeling of SAAP-148 with TAMRA reduced the efficacy of the peptide towards planktonic GFP-producing MRSA (Figure S6). For confocal microscopy, a suboptimal concentration (5 µM) of TAMRA-SAAP-148 was used to allow visualization of both peptide and bacteria. Results revealed that redispersed PVA-lyophilized TAMRA-SAAP-148 nanogels seemed to partly eradicate MRSA from the superficial layers of the model to the same extend as TAMRA-SAAP-148, as indicated by the reduction in MRSA-GFP fluorescent signal compared to PBS (Figure 5b–g). In addition, in both cases TAMRA-SAAP-148 was internalized in a substantial number of cells in these superficial cell layers. Moreover, it was observed that TAMRA-SAAP-148 and MRSA co-localized, confirming a MRSA-bound fraction of TAMRA-SAAP-148. Together, these data indicate that both MRSA and TAMRA-SAAP-148 localize in the superficial cell layers, but that the capability of TAMRA-SAAP-148 nanogels to penetrate deeper layers of the 3D human epidermal model is limited.

## 4. Discussion

Antimicrobial peptides such as SAAP-148 are promising alternatives to current antibiotics for the treatment of bacterial wound infections. However, the cutaneous use of SAAP-148 is hampered by limitations related to its peptidic nature resulting in low bioavailability in the wound environment due to protein binding and/or degradation by proteases along with cytotoxicity due to its cationic nature, altogether resulting in a narrow therapeutic window [10,12]. It is hypothesized that these limitations can be overcome by using nanogels as a drug delivery system. Here, we describe the use of a nanogel formulation for cutaneous application with the aim to decrease cytotoxicity, while maintaining antimicrobial activities, thereby increasing the selectivity index of SAAP-148.

OSA-HA nanogels encapsulating 150, 175 and 200 µg/mL of SAAP-148 were prepared and characterized. These anionic nanogels ranged from 229 to 419 nm in size, with increased SAAP-148 concentrations being positively correlated with increased nanogel sizes. The use of microfluidics allowed precise control during the production of the nanogels [34], thus resulting in a low polydispersity of the nanogel solutions. All SAAP-148-loaded nanogels showed a more neutral ZP compared to placebo nanogels in accordance with previous reports [16,35], suggesting the presence of surface-bound peptide in addition to encapsulated peptide. Moreover, the cationic SAAP-148 was very efficiently encapsulated in these anionic nanogels, resulting in a high DL of more than 20% (*w*/*w*). This DL is much higher than that achieved for most existing nanomedicines (DL < 10%) [36], which further emphasizes the potential of OSA-HA nanogels as high AMP-loading nanomedicine. Lyophilization of these nanogels using 10 mg/mL PVA maintained their optimal physicochemical properties upon reconstitution in ultrapure water, while dextran-40 was not effective in maintaining the small size of the nanogels. These findings contrast with those of our previous lyophilization study of OSA-HA nanogels loaded with snake cathelicidin Ab-Cath [van Gent and Nibbering, personal communication], where both PVA and dextran-40 were effective cryoprotectants. We hypothesize that the lower molecular weight, lower net charge and higher hydrophilicity of SAAP-148 compared to Ab-Cath could potentially explain the suboptimal protection of SAAP-148-loaded nanogels by dextran-40.

It is hypothesized that release of peptides from nanogels such as OSA-HA is triggered by the presence of salts, changes in pH and/or by degradation of the polymer [22]. In this study, release of SAAP-148 from OSA-HA nanogels was evaluated in PBS and by using the dialysis method it was revealed that SAAP-148 was released in a sustained manner reaching a total release of 37–41% of SAAP-148 within 72 h, as was also demonstrated to be the maximum released amount found by using the centrifugation method. Contradictorily, using the centrifugation method this fraction of SAAP-148 was released immediately, which is most likely the effect of physical stress during centrifugation that forces not tightly bound SAAP-148 out of the nanogel. The release rate of SAAP-148 from OSA-HA nanogels was slower than that of DJK-5 with 80% release in 5 h and complete release in 48 h [16], but very comparable to that of novicidin with 55% release in 72 h followed by a sustained release phase over 12 days [27]. Moreover, in 72 h, SAAP-148-loaded nanogels doubled in size and became more neutrally charged, indicating only partial dissociation of the nanogels. Therefore, we believe that SAAP-148 release from OSA-HA nanogels is triggered by presence of PBS, but that approximately 60% of SAAP-148 remains associated with the HA polymer likely due to strong electrostatic and hydrophobic interactions. The possibility of SAAP-148 binding to the dialysis membrane or plastic was taken into account by correcting for the total recovery of SAAP-148 solution, which ranged from 65–96% (Figure S4). Nevertheless, the maintained antimicrobial activity of SAAP-148-loaded nanogels at 24 h exposure in combination with 30–41% SAAP-148 released in this time period implies that the HA-associated SAAP-148 fraction remains antimicrobial.

The capability of these OSA-HA nanogels to shield the cationic charge of SAAP-148 resulting in anionic nanogels is important to improve the safety of this peptide, since an increase in the net charge of such peptides has been linked to increased hemolysis and/or cytotoxicity to eukaryotic cells [37,38]. The improved safety of SAAP-148 upon encapsulation in OSA-HA nanogels was confirmed for both 4 h and 24 h of exposure to a range of eukaryotic cells present in the wound environment, i.e., human erythrocytes, human primary skin fibroblasts and human keratinocytes. This is in line with previous studies indicating that OSA-HA nanogels are particularly effective at shielding the cationic charges of encapsulated peptides [27,28]. Moreover, the sustained release of SAAP-148 from OSA-HA nanogels could play a role in the reduced cytotoxicity upon formulation, as cells might tolerate exposure to increasing concentrations of peptide released from the nanogels over time compared to the total dose directly available when using the peptide in solution. Indeed, the improved safety of SAAP-148-loaded nanogels compared to SAAP-148 is most pronounced (2.2–3.6-fold) at 4 h exposure but is still observed at 24 h (1.5–2.9-fold), and can be related to the sustained release of SAAP-148 (22–39% at 4 h; 30–41% at 24 h). Notably, the enhanced safety was also proven in a 3D human epidermal model that is more resistant to cytotoxic activities of SAAP-148 [29].

Importantly, the antimicrobial activities against planktonic, biofilm-residing and skin-colonized AMR *S. aureus* and *A. baumannii* were maintained for SAAP-148-loaded OSA-HA nanogels, but because of its sustained release required up to 24 h of exposure to reach the same antimicrobial activity as SAAP-148. For future studies, it would be of interest to investigate whether these effects also hold for polymicrobial communities of *S. aureus* and *A. baumannii*, as co-infections are very common in diabetic wounds [39]. Together, the improved safety combined with maintained antimicrobial activity resulted in an up to 2.9-fold improvement in the selectivity index for SAAP-148 when encapsulated in OSA-HA nanogels. A similar improvement in selectivity index was previously reported for the peptides DJK-5, LBP-3 and novicidin after encapsulation in OSA-HA nanogels [16,27,28], further emphasizing the applicability of this delivery system for a range of AMPs. Potentially, this selectivity index could be further improved by actively targeting the bacteria and/or site of infection. This could be achieved by functionalization of the HA polymer with ligands of the bacterial surface [40] or by rendering the nanogel responsive to stimuli of the bacterial microenvironment, such as pH changes or presence of enzymes and molecules associated with bacterial infections [41].

Markedly, confocal microscopy showed that GFP-producing MRSA infected the 3D human epidermal model only in the superficial layers and that TAMRA-SAAP-148 nanogels located in the same layers, indicating favorable localization of TAMRA-SAAP-148 to eradicate the bacterial skin infection. Notably, F-actin breakdown and DNA degradation is part of the keratinocyte differentiation process occurring in the 3D human epidermal model [42], which explains the suboptimal staining of the superficial layers (e.g., stratum corneum) in the model. Moreover, it is important to emphasize that the GFP-labeled MRSA is genetically different from the AMR *S. aureus*, although they were similarly susceptible to SAAP-148 (Figure S6). Their differences in infection efficiency and penetration capability were not further investigated in the present study. In addition, labeling of SAAP-148 with TAMRA reduced the efficacy of the peptide towards the GFP-producing MRSA from 6.4 µM to 25.6 µM (Figure S6). This was expected, as reduced antimicrobial activities of AMPs upon fluorescently labeling have been reported previously [43]. Together, confocal microscopy revealed that TAMRA-SAAP-148 nanogels as well as TAMRA-SAAP-148 did not penetrate deeper layers of the model. In the future, using a wounded skin model should be considered, as used by others [44,45,46], which might facilitate penetration of SAAP-148-loaded nanogels into deeper layers of the tissue and allow for studying wound healing processes.

## 5. Conclusions

In conclusion, OSA-HA nanogels are a promising delivery system for cutaneous application of SAAP-148 to treat skin wound infections. SAAP-148-loaded nanogels exhibited excellent physicochemical properties, including a small size (219 nm), low polydispersity (PDI = 0.03), anionic surface charge (ZP = −14.5 mV), high encapsulation efficiency (98.7%) and drug loading capacity (22.8%) and a sustained release profile (37–41% released in 72 h). Moreover, OSA-HA nanogels maintained the antimicrobial activity of SAAP-148 against AMR bacterial strains, while reducing its cytotoxicity against eukaryotic cells present in the wound environment >2-fold, thus improving the selectivity index of SAAP-148 up to 2.9-fold in conditions relevant for skin wound infections. Although the nanogels showed excellent performance using in vitro models, SAAP-148′s proteolytic stability after encapsulation remains to be investigated using proteases. Moreover, more extensive evaluation using in vivo models would allow for a better understanding of the delivery system’s pharmacokinetic and -dynamic properties. Additionally, development of a macroscale delivery system, such as a gel, cream or ointment, containing the SAAP-148-loaded OSA-HA nanogels would help assess the potential of this nanoscale delivery system as an antimicrobial treatment for clinical applications.

## 6. Patents

The SAAP-148 peptide described in the present study is patented by the Leiden University Medical Center (patent number WO2015088344; the co-inventors are J.W.D. and P.H.N.).

## Figures and Tables

**Table 1:** Physicochemical properties of freshly produced OSA-HA nanogels prepared with increasing amounts of SAAP-148.

SAAP-148 (µg/mL)	OSA-HA (µg/mL)	Size (nm)	PDI	ZP (mV)	EE (%)	DL (%)
0	500	226 ± 11	0.16 ± 0.04	−32.3 ± 3.4	-	-
150	500	229 ± 24	0.03 ± 0.01	−14.5 ± 1.2	98.7 ± 0.4	22.8 ± 0.1
175	583	295 ± 32 *	0.05 ± 0.00	−14.6 ± 0.9	98.9 ± 0.5	22.9 ± 0.1
200	667	419 ± 80 *	0.04 ± 0.03	−12.3 ± 1.3	99.0 ± 0.6	22.9 ± 0.1

Data are mean ± SD of 3-16 independent sample batch replicates. * Significantly different (*p* = 0.0021) from nanogels prepared with 150 µg/mL SAAP-148. Abbreviations: OSA-HA = octenyl succinic anhydride-modified hyaluronic acid; SAAP-148 = synthetic antimicrobial and antibiofilm peptide 148; PDI = polydispersity index; ZP = zeta potential; EE = encapsulation efficiency; DL = drug loading.

**Table 2:** Physicochemical properties of redispersed SAAP-148-loaded and placebo OSA-HA nanogels upon lyophilization in presence of the cryoprotectants PVA or dextran-40.

	Lyophilized, Cryoprotectant	Size (nm)	PDI	ZP (mV)	EE (%)	DL (%)
**SAAP-148-loaded nanogel (150 µg/mL)**	No	229 ± 24	0.03 ± 0.01	−14.5 ± 1.2	98.7 ± 0.4	22.8 ± 0.1
	Yes, none	435 ± 38	0.80 ± 0.35	−24.4 ± 0.2	82.0 ± 1.0	19.7 ± 0.2
	Yes, 1 mg/mL PVA	306 ± 6	0.67 ± 0.35	−29.5 ± 0.4	90.6 ± 1.0	21.4 ± 0.2
	Yes, 5 mg/mL PVA	294 ± 27	0.13 ± 0.01	−30.1 ± 0.7	86.1 ± 2.9	20.5 ± 0.5
	Yes, 10 mg/mL PVA	262 ± 53 *	0.12 ± 0.00	−29.9 ± 0.6	93.4 ± 2.2	21.9 ± 0.4
	Yes, 1 mg/mL dextran-40	433 ± 56	0.88 ± 0.21	−25.2 ± 0.2	90.9 ± 1.0	21.4 ± 0.2
	Yes, 5 mg/mL dextran-40	379 ± 4	0.35 ± 0.06	−25.6 ± 0.2	88.2 ± 7.8	20.9 ± 1.5
	Yes, 10 mg/mL dextran-40	388 ± 20	0.24 ± 0.04	−25.0 ± 0.2	91.6 ± 2.0	21.6 ± 0.4
**Placebo nanogel**	No	226 ± 11	0.16 ± 0.04	−32.3 ± 3.4	-	-
	Yes, 10 mg/mL PVA	284 ± 8	0.68 ± 0.05	−25.6 ± 3.1	-	-

Data are presented as mean ± standard deviation of 3–16 independent sample batch replicates. * Non-significant difference compared to fresh SAAP-148-loaded nanogels. Abbreviations: OSA-HA = octenyl succinic anhydride-modified hyaluronic acid; SAAP-148 = synthetic antimicrobial and antibiofilm peptide 148; PVA = polyvinyl alcohol; PDI = polydispersity index; ZP = zeta potential; EE = encapsulation efficiency; DL = drug loading.

**Table 3:** Bacterial activities of redispersed PVA-lyophilized SAAP-148-loaded nanogels on planktonic and biofilm-residing AMR *S. aureus* and *A. baumannii* compared to SAAP-148 and placebo nanogel.

			LC_99.9_ or BEC_99.9_ (µM)		
Species	Strain	Exposure	SAAP-148	SAAP-148 Nanogel	Placebo Nanogel
	Planktonic bacteria				
*S. aureus*	LUH14616 RUH875	4 h	**6.4**	**12.8**	**>102.4**
		24 h	**6.4**	**6.4**	**>102.4**
*A. baumannii*		4 h	**6.4**	**12.8**	**>25.6**
		24 h	**6.4**	**6.4**	**>25.6**
	Biofilm-residing bacteria				
*S. aureus*	LUH14616 RUH875	24 h	**51.2**	**51.2**	**>204.8**
*A. baumannii*		24 h	**51.2** (51.2–102.4)	**102.4**	**>204.8**

Planktonic bacteria were exposed for 4 h or 24 h to SAAP-148, SAAP-148-loaded nanogel or placebo nanogel in PBS in presence of 50% (*v*/*v*) plasma. Twenty-four-hour immature biofilms were exposed for 24 h to SAAP-148, SAAP-148-loaded nanogel or placebo nanogel in PBS. Data are expressed as lethal concentration (LC)_99.9_, i.e., the lowest SAAP-148 concentration killing 99.9% of planktonic bacteria, or as biofilm eradication concentration (BEC)_99.9_, i.e., the lowest SAAP-148 concentration eliminating 99.9% of the biofilm-residing bacteria. Results are shown as median (bold) and range if applicable (in brackets) of three independent experiments performed in duplicate.

**Table 4:** Hemolytic and cytotoxic activities of PVA-lyophilized SAAP-148-loaded nanogels redispersed in biorelevant medium compared to SAAP-148 and placebo nanogel.

	Hemolytic Activity or Cytotoxicity (µM)		
Exposure	SAAP-148	SAAP-148 Nanogel	Placebo Nanogel
Human erythrocytes			
1 h (PBS)	**6.9** (6.4–7.3)	**13.1** (7.5–15.6)	**>51.2**
1 h (50% plasma)	**160.1** (156.7–267.8)	**400.5** (270.6–474.8)	**>204.8**
Human primary skin fibroblasts			
4 h	**6.0** (4.9–6.1)	**21.4** (12.3–28.0)	**>51.2**
24 h	**4.4** (3.2–5.3)	**12.9** (12.5–28.0)	**>51.2**
Ker-CT keratinocytes			
4 h	**2.2** (1.8–2.9)	**5.0** (4.3–9.2)	**>51.2**
24 h	**2.6** (2.2–2.8)	**4.0** (3.6–9.4)	**>51.2**

Values presented are cytotoxicity values of (i) 2% (*v*/*v*) human erythrocytes in PBS or 50% (*v*/*v*) plasma after 1 h exposure to SAAP-148 solution or nanogels, (ii) a monolayer of human primary skin fibroblasts in DMEM medium with 0.5% (*v*/*v*) human serum after 4 h and 24 h exposure to SAAP-148 solution or nanogels, and (iii) a monolayer of Ker-CT keratinocytes in KSFM medium after 4 h and 24 h exposure to SAAP-148 solution or nanogels. Results are depicted as median (bold) and range (in brackets) of the EC_50_, i.e., the calculated concentration of the agents resulting in 50% cytotoxicity of three independent experiments performed in triplicate.

## Data Availability

The data presented in this study are available within the article or are available on request from the corresponding author.

## Supplementary Materials

The following supporting information can be downloaded at: https://www.mdpi.com/article/10.3390/pharmaceutics15020429/s1, Figure S1: Reaction scheme of modification of hyaluronic acid (HA) with octenyl succinic anhydride (OSA), resulting in OSA-modified HA (OSA-HA); Figure S2: ^1^H-NMR of OSA-HA batch with rate of substitution of 15%; Figure S3: ^1^H-NMR of OSA-HA batch with rate of substitution of 20%; Figure S4: Release profile of SAAP-148 solution; Figure S5: Cross-section of a non-colonized 3D human epidermal model; Figure S6: In vitro killing of AMR *S. aureus* LUH14616 and GFP-producing MRSA upon 4 h exposure to SAAP-148 or TAMRA-SAAP-148 in 50% plasma.
