# Supplementary material for: Encapsulation of SAAP-148 in Octenyl Succinic Anhydride-Modified Hyaluronic Acid Nanogels for Treatment of Skin Wound Infections
Source: Pharmaceutics. 2023 Jan 28;15(2):429. doi: 10.3390/pharmaceutics15020429 (PMC9967827; doi:10.3390/pharmaceutics15020429)
Supplement: Supplementary file 1 [file pharmaceutics-15-00429-s001.zip › pharmaceutics-2135898-supplementary.pdf]

Supplementary Materials

Encapsulation of SAAP-148 in Octenyl Succinic Anhydride-Modified Hyaluronic Acid Nanogels for Treatment of Skin Wound Infections

Miriam E. van Gent [1,*], Tom van Baaren [1], Sylvia N. Kłodzińska [2], Muhanad Ali [1], Natasja Dolezal [3], Bjorn R. van Doodewaerd [4], Erik Bos [5], Amy M. de Waal [1], Roman I. Koning [5], Jan Wouter Drijfhout [3], Hanne M ørck Nielsen [2] and Peter H. Nibbering [1]

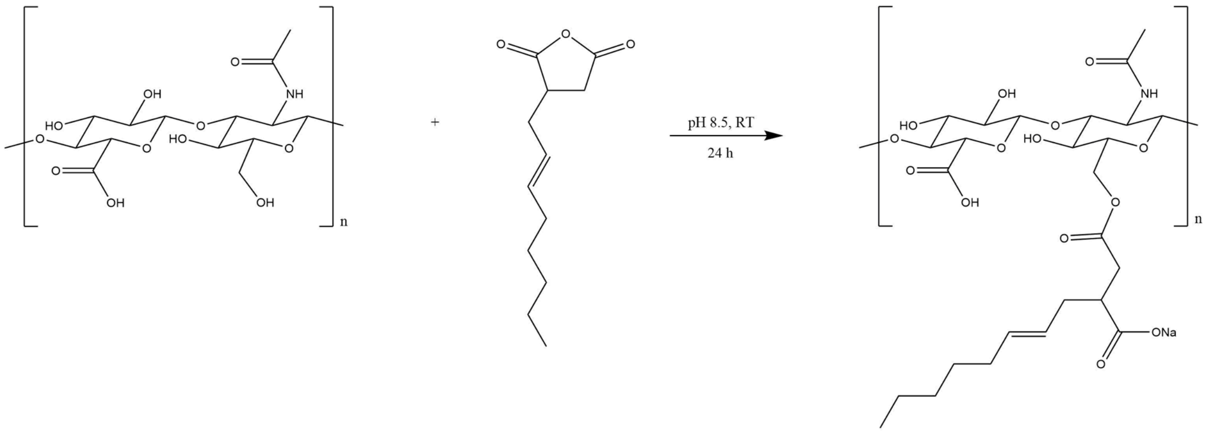

Figure S1. Reaction scheme of modification of hyaluronic acid (HA) with octenyl succinic anhydride (OSA), resulting in OSA-modified HA (OSA-HA). Reaction was conducted in $NaHCO_3$-buffered solution (2.0 M) adjusted to pH 8.5 for 24 h at room temperature.

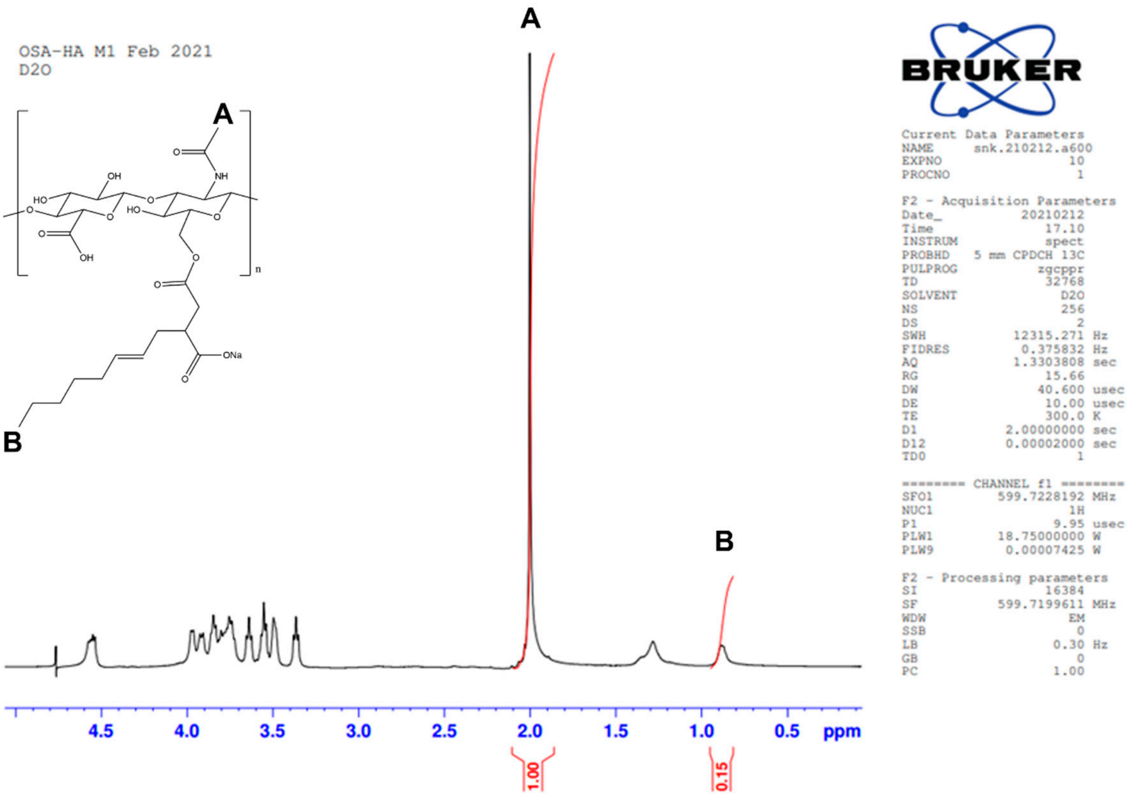

Figure S2. ^{1}H-NMR of OSA-HA batch with rate of substitution of 15%. This spectrum was acquired in D_2O at 25°C and 400 MHz and at an OSA–HA concentration of 10 mg/mL. Peaks were assigned according to Eenschoten et al [1].

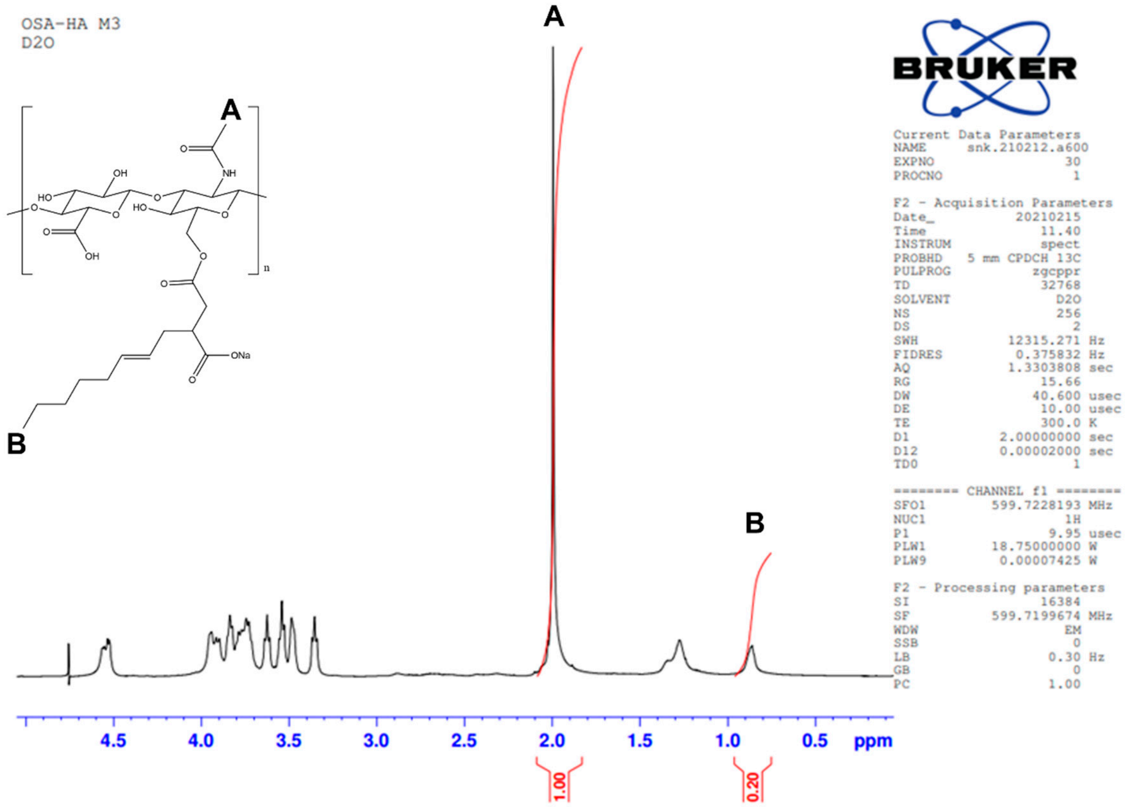

Figure S3. ^{1}H-NMR of OSA-HA batch with rate of substitution of 20%. This spectrum was acquired in D_2O at 25°C and 400 MHz and at an OSA–HA concentration of 10 mg/mL. Peaks were assigned according to Eenschoten et al [1].

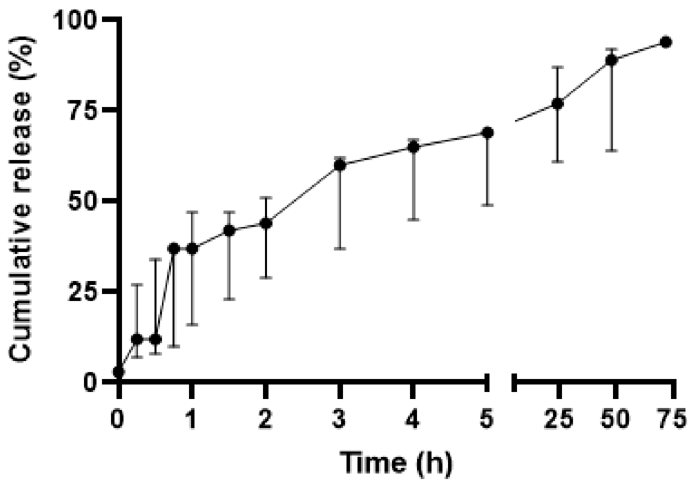

Figure S4. Diffusion of SAAP-148 from the float-a-lyzer casette to the receiver medium. Release of SAAP-148 solution in PBS at 37°C using the float-a-lyzer dialysis method. Data are presented as median and error of three independent experiments. Data is normalized to the maximum amount of SAAP-148 recovered during each experiment, which ranged from 65-96%.

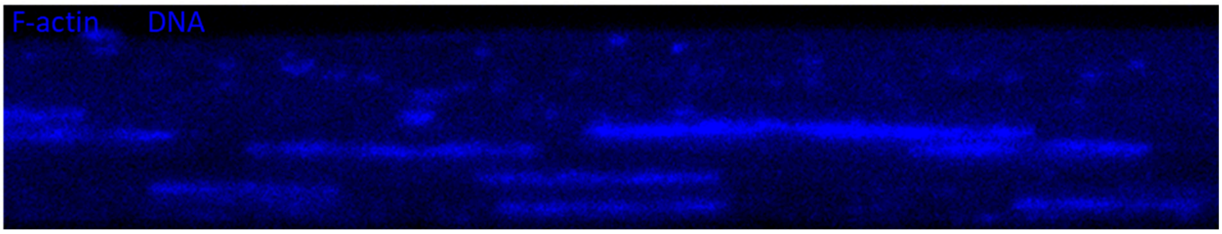

Figure S5. Cross-section of a non-colonized 3D human epidermal model. Fluorescence confocal microscopy of a control 3D human epidermal model treated for 4 h with PBS. Thickness of the 3D model was roughly 25 µM. Samples were stained for filamentous actin (F-actin) using AlexaFluor 405 (blue) and with 4′,6-diamidino-2-phenylindole (DAPI) DNA staining (blue). Images are taken with a 63× oil lens and shown is a representative image of two experiments performed in duplicate.

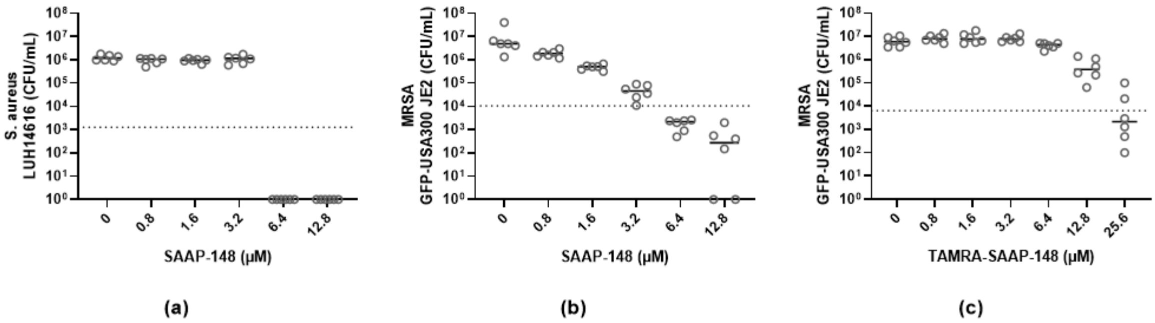

Figure S6. *In vitro* killing of AMR *S. aureus* LUH14616 and GFP-producing MRSA upon 4 h exposure to SAAP-148 or TAMRA-SAAP-148 in 50% plasma. Ability of SAAP-148 to eradicate (a) AMR *S. aureus* LUH14616 and (b) GFP-producing MRSA and the ability of TAMRA-SAAP-148 to eradicate (c) GFP-producing MRSA upon 4 h exposure in 50% plasma. Results are expressed as median and individual values of three experiments performed in duplicate. The dashed line indicates the $LC_{99.9}$, i.e. the lowest SAAP-148 concentration required to eradicate 99.9% of the planktonic bacteria.

References

1. Eenschooten, C., et al., Preparation and structural characterisation of novel and versatile amphiphilic octenyl succinic anhydride-modified hyaluronic acid derivatives. *Carbohydrate Polymers*, **2010**. *79*(3): p. 597-605.
